# Photoaffinity labeling of transcription factors by DNA-templated crosslinking†
†Electronic supplementary information (ESI) available: Experimental details, characterization data, DNA sequences, and detailed selection procedure. See DOI: 10.1039/c4sc01953a



**DOI:** 10.1039/c4sc01953a

**Published:** 2014-10-01

**Authors:** Ying Liu, Wenlu Zheng, Wan Zhang, Nan Chen, Yang Liu, Li Chen, Xiaozhou Zhou, Xingshuo Chen, Haifeng Zheng, Xiaoyu Li

**Affiliations:** a Key Laboratory of Bioorganic Chemistry and Molecular Engineering of the Ministry of Education , Beijing National Laboratory of Molecular Sciences , College of Chemistry and Molecular Engineering , Peking University , Beijing , China 100871 . Email: xiaoyuli@pku.edu.cn; b Key Laboratory of Chemical Genomics , School of Chemical Biology and Biotechnology , Peking University Shenzhen Graduate School , Shenzhen , China 518055

## Abstract

Characterization of transcription factor-DNA interaction is of high importance in elucidating the molecular mechanisms of gene transcriptions. DNA-based affinity probes were developed to capture and identify transcription factors by covalent crosslinking; however, the requirement of a crosslinker on the affinity probe remains a disadvantage, as the crosslinker itself often interferes with the protein–DNA interactions. We report a dual-probe method able to capture DNA-binding transcription factors with unmodified protein-binding sites in scenarios where conventional probes have failed. We have also shown the method's converse application in selecting specific transcription factor-binding DNA sequences from a probe library and its extension to studying proteins recognizing epigenetic marks. This study may provide a new tool for exploring DNA-binding proteins in biology.

## Abstract 2

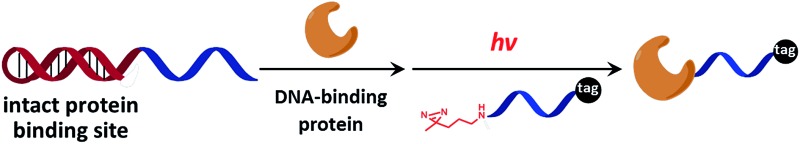
A dual-probe system can specifically capture DNA-binding proteins with an unmodified binding site.

## Introduction

Transcription factor (TF) is the major class of DNA-binding proteins that recognize and bind to specific double strand DNA sequences.^1–3^ By binding to DNA, transcription factors modulate transcription levels of target genes and play central roles in many fundamental biological processes, usually in response to various exogenous and endogenous cellular signals in both healthy and disease states.^1,4–7^ Consequently, transcription factors have been intensively pursued as drug targets in pharmaceutical research.^8–11^

Characterization of TF–DNA interactions is instrumental in elucidating transcription factors' regulatory mechanisms. Previously, many methods have been developed to identify known transcription factors' binding DNA sequences,^12^ such as footprinting,^13,14^ electrophoresis mobility shift assay (EMSA),^15,16^ chromatin immunoprecipitation (ChIP),^17^ chemiluminescent pull-down assay,^18,19^ protein binding microarray,^20^ and HT-SELEX.^21^ Once a transcription factor's binding DNA sequence is known, it can be embedded into various probes for sensitive detection, such as the bimolecular proximity assay,^22–25^ proximity-ligation assay,^26,27^ nuclease protection assay,^28^ transcription factor beacon,^29^ fluorescence recovery assay,^30^ and enzyme amplification assay.^31^

**Fig. 1:**
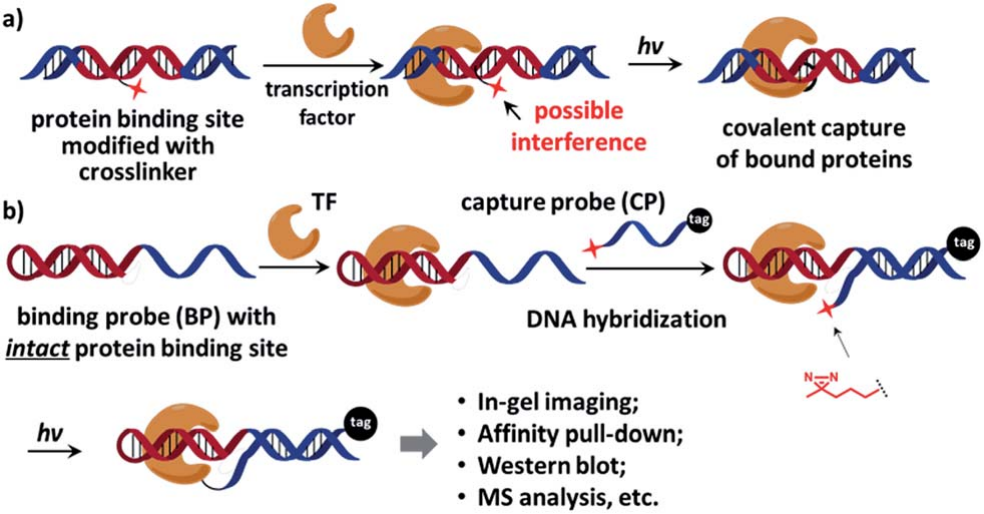
(a) The crosslinker (shown as a red star) inside the protein-binding site of the affinity probe may interfere with protein binding. (b) The dual-probe method: after the transcription factor binds the binding probe (BP), the capture probe (CP) hybridizes to the BP DNA, photo-crosslinks BP-bound proteins under light irradiation, and also tags the protein. See the ESI† for probe structures and synthesis details.

On the other hand, characterization of unknown transcription factors that bind to specific DNA sequences is also highly important.^32,33^ As many transcription factors bind to DNA transiently with low affinity,^7^ the interactions are often lost during typical affinity purification; therefore covalent affinity probes equipped with chemical and photo-crosslinkers were developed, serving as a powerful tool to study protein–DNA interactions.^14,34–51^ However, since the crosslinker is usually located in the protein-binding site of the affinity probe, it often contributes to or interferes with protein binding (Fig. 1a). The probe's performance strongly depends on the nature and position of the crosslinker.^38,41,42^ Considerable efforts were undertaken to minimize the crosslinker's impact by screening for the optimal position,^41,43,49,52^ adjusting the crosslinker's orientation,^46,47,52–54^ and using smaller crosslinking groups.^47^ In a recent report, Famulok and co-workers conjugated the crosslinker at the end of the aptamer so that binding interference was avoided.^40^ Indeed, ideally an affinity probe should contain a protein-binding site free of modifications, but is still able to specifically deliver the crosslinker to the proximity of the target protein for effective labeling.

Recently we reported an affinity labeling method for identifying small molecule's target proteins, in which the functions of target recognition and covalent crosslinking are separated into two probes.^55,56^ We reason that this strategy may be employed in studying transcription factor-DNA interactions to circumvent the requirement for a crosslinker within the affinity probe. Our design is shown in Fig. 1b, a native, modification-free hairpin DNA containing the bait sequence (shown in red) is used as the “binding probe” (BP). Another DNA modified with a photoreactive 3′-diazirine group serves as the “capture probe” (CP), which also bears a 5′-tag customizable for subsequent analysis (*e.g.* a fluorophore for in-gel imaging or a biotin group for affinity pull-down). Diazirine has been widely used as the crosslinker in numerous biological applications for its small size, high reactivity, and biocompatibility;^47,57–60^ it also exhibits very low non-specific protein crosslinking with moderately elevated salt concentration.^55,56^ After the transcription factor binds to BP, CP hybridizes to the binding probe DNA and then photo-crosslinks BP-bound protein under light irradiation. BP is free of any modification so that the original protein–DNA interaction is maintained, while CP is able to deliver the crosslinker close to the target protein for efficient crosslinking.

## Results and discussion

**Fig. 2:**
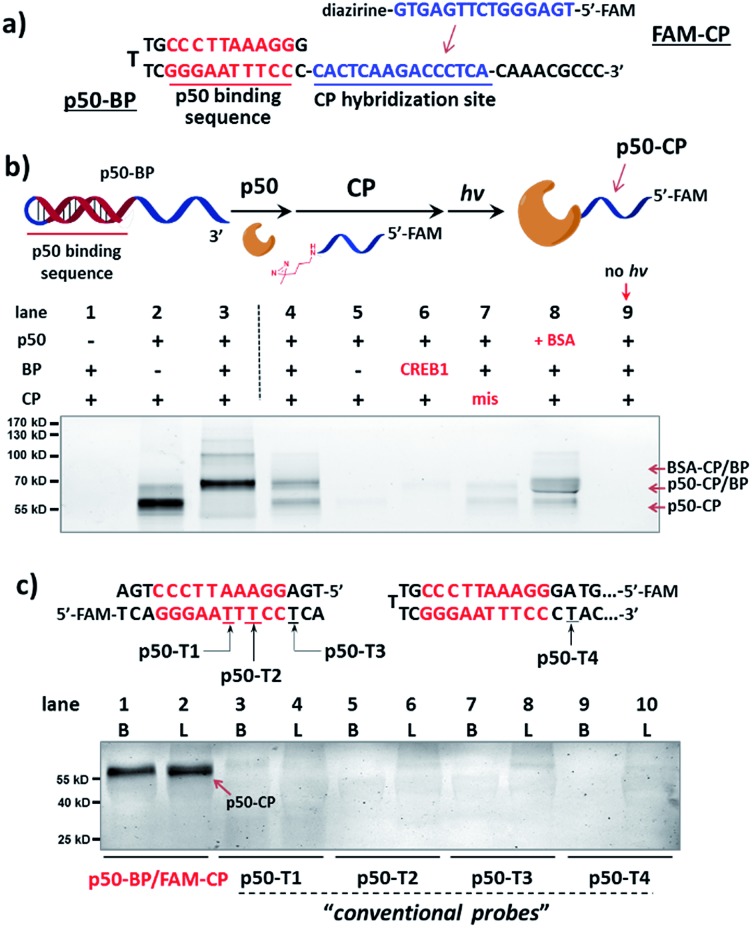
(a) Sequences of p50-BP and FAM-CP. (b) Reaction scheme and denaturing SDS-PAGE analysis, monitored by FAM fluorescence. p50-BP, FAM-CP, p50, and BSA: 2 μM each; *hv*: 365 nm, 15 min, 0 °C. Buffer: 10 mM MgCl_2_, 50 mM KCl, 10 mM EDTA, 25 mM DTT, 1× PBS. Lane 1: BP/CP only; lane 2: standard sample of the p50-CP conjugate; lane 3: standard sample of the DNA duplex formed by p50-CP and BP; lane 4: p50 capture by p50-BP/FAM-CP after irradiation; lane 5–9: same as lane 4 but without p50-BP, with a non-p50-binding (CREB1-binding) BP, with a sequence-mismatched FAM-CP, with 1 eq. additional BSA, and without irradiation. p50-conjugated CP and p50-BP may partially renature in gel, resulting in two fluorescent bands: the p50-CP conjugate and the p50-CP/BP duplex (marked by arrows).^55^ (c) Comparison of p50 capture by the dual-probe method with probes having directly conjugated diazirine (p50-T1/2/3/4). Experimental conditions are the same as in (b). Diazirine sites are underlined. B: reactions in buffer; L: reactions in HeLa lysate (4.8 mg mL^–1^, spiked with 2 μM p50). CREB1: cAMP response element-binding protein 1.

We initiated the study with a model transcription factor p50, a subunit of nuclear factor-kappa B (NF-*κ*B) transcription factor,^61^ which plays key roles in cell's immune responses to stimuli^62^ and is implicated in many diseases.^63^ First, a binding probe embedded with the p50-binding sequence (p50-BP) and a sequence-complementary capture probe with a 5′-fluorescein tag (FAM-CP) were prepared (Fig. 2a and see details in Fig. S1†). The mixture of p50-BP, FAM-CP, and p50 was irradiated under 365 nm before denaturing SDS-PAGE analysis (Fig. 2b). Results show that p50 can be specifically labelled by the dual probe (lane 4). Two product bands were observed in lane 4: by comparing with the standard samples in lane 2 and 3, they are considered to be the p50-CP conjugate (p50-CP, lower band) and the DNA duplex formed by the p50-CP and BP DNA (p50-CP/BP, upper band), as a DNA duplex may partially renature in SDS-PAGE. We have observed and experimentally confirmed this phenomenon previously.^55^ Furthermore, little non-specific labeling was observed when additional BSA was added along with the p50 protein (1 eq. in lane 8 of Fig. 2c; 10 eq. in lane 8 and 9 of Fig. S5†). Other negative controls (without p50-BP, with a CREB-1-binding BP, and without light irradiation; lane 5, 6, and 9) also did not give noticeable p50 capture. An FAM-CP with mismatched DNA sequence for the p50-BP showed some low level of labeling, possibly resulting from the BP-CP duplex partially formed at the incubation temperature (0 °C). In addition, similar labeling specificity was also observed with a 5′-biotin-tagged CP (Fig. S5†). Collectively, these results have demonstrated that the observed p50 labeling requires both specific protein–DNA interaction and photo-crosslinking mediated by a complementary capture DNA probe.

**Fig. 3:**
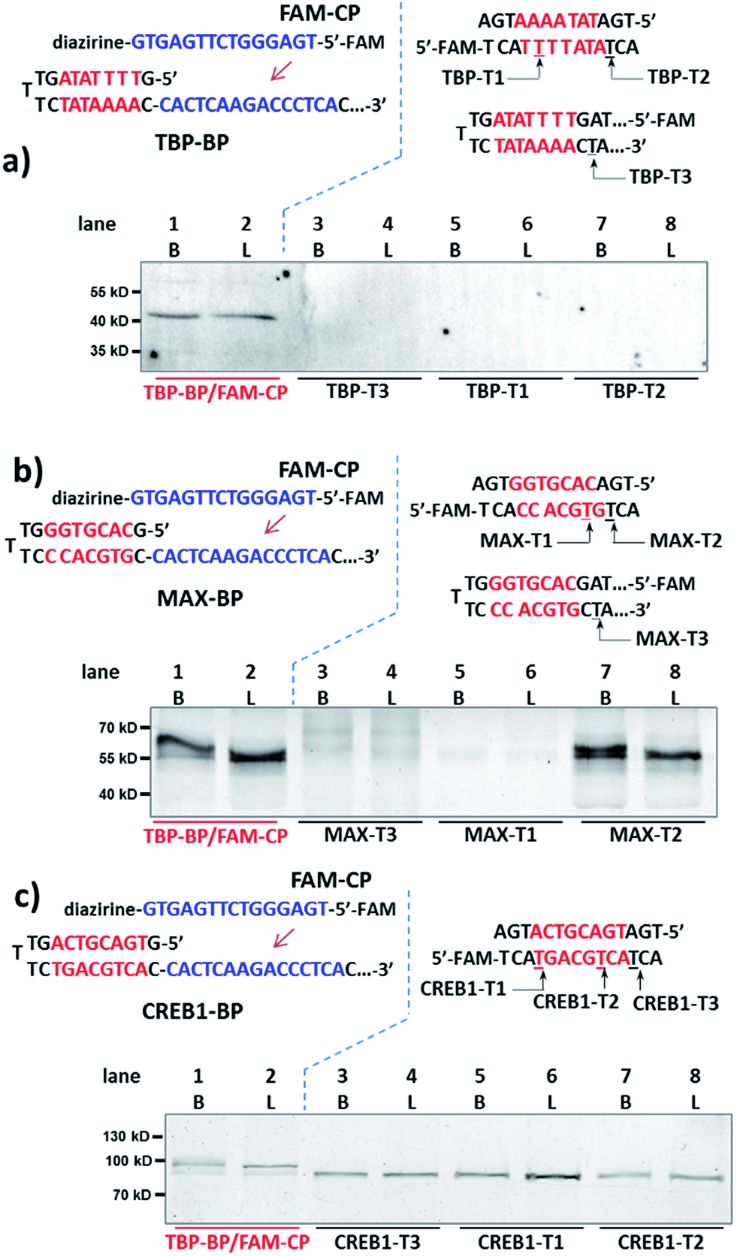
Binding probe and capture probe sequences and protein labeling results analysed by denaturing SDS-PAGE for (a) TBP, (b) MAX and (c) CREB1. Reaction procedure and conditions are the same as in Fig. 2b and c, except that no BSA was added. Diazirine sites are underlined. B: reactions in buffer; L: reactions in HeLa lysate (4.8 mg mL^–1^, spiked with 2 μM transcription factor protein).

Next, for comparison, a series of “conventional probes” were prepared with the diazirine crosslinker directly conjugated at the major groove side of the DNA duplex (Fig. S2†), either inside the p50-binding site (p50-T1, T2), immediately next to it (p50-T3), or 1-base away from the binding site (p50-T4; Fig. 2c). These probes were subjected to the same p50 labeling procedures as in Fig. 2b. However, in contrast to the dual probe, none of these affinity probes was able to effectively capture p50, either in buffer, in cell lysates, or in nuclear extracts (Fig. 2c & S6†). Intrigued by this result, we further tested more transcription factors: TATA-binding protein (TBP),^64^ Myc-associated factor X (MAX),^65^ and CREB1.^66^ Matching pairs of TF-BP/FAM-CP and several series of “conventional probes” were prepared for each transcription factor respectively (Fig. 3). These probes were subjected to the same labeling procedures as in Fig. 2 and their performances were compared. First, all pairs of BP/CPs can capture their respective protein targets (Fig. 3; lane 1 and 2) and also showed specificity similar to the p50 probes (Fig. S7†). Interestingly, although TBP is known to primarily interact with DNA's minor groove,^67^ none of the “conventional probes” (with the crosslinker in the major groove) showed detectable labeling (Fig. 3a; lane 3–8). However, MAX-T2, which has the diazirine crosslinker immediately next to the binding site, was able to capture the MAX protein (Fig. 3b), and MAX-T1 and T3, with the diazirine inside and away from the binding site respectively, showed very little MAX capture. Although MAX and CREB1 are both leucine zipper family proteins and they bind DNA's major groove very similarly,^68,69^ all CREB1 probes can capture the CREB1 protein.

**Fig. 4:**
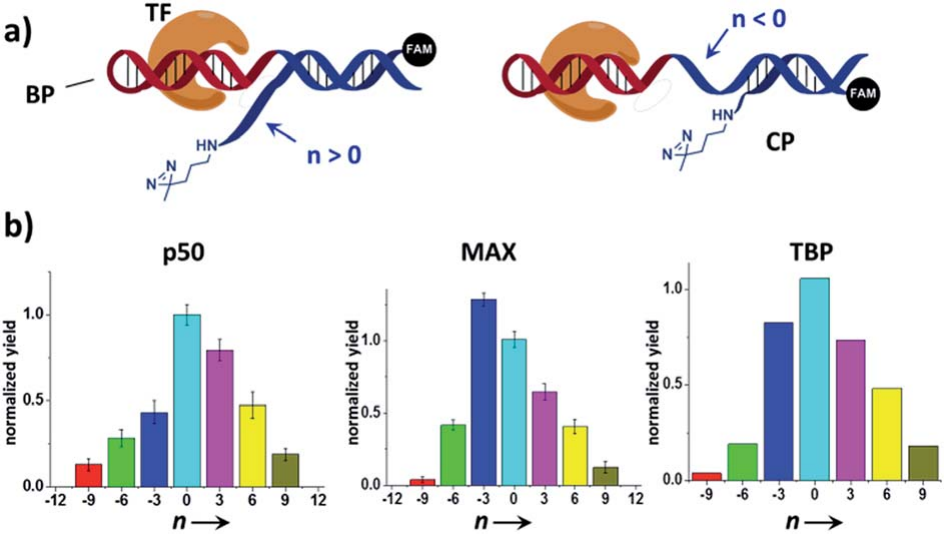
(a) Varying the BP/CP hybridization site to change the diazirine position relative to the transcription factor target. “*n*” denotes the number of protruding (*n* > 0) or recessing (*n* < 0) nucleobases after probe hybridization. (b) BP/CP pairs of different *n* values were subjected to the same protein labeling procedure as in Fig. 2 for p50, MAX, and TBP. Labeling yields were determined by measuring the TF-CP fluorescence in denaturing SDS-PAGE and normalized to *n* = 0. Error bars (SD) in the p50 and MAX experiments are based on three separate experiments. The TBP experiments were performed only once.

We reason that there may be two possible underlying reasons for these observations: (i) the diazirine crosslinker may have sterically hindered the protein binding, as suggested by several crystal structures of TF-DNA complexes;^70,71^ (ii) the specific structure and conformation of the “conventional probes” do not allow for a productive crosslinking (*e.g.*, the linker connecting the diazirine to DNA may be too short or lack sufficient flexibility).^43,52^ With the dual-probe method, the crosslinker may have better flexibility and its spatial position can be feasibly varied to access the protein target without having to be part of the binding probe. In order to test this, we compared the labeling of p50, MAX, and TBP with BP/CP pairs having different “*n* values” (*n* represents the number of protruding or recessing nucleobases after BP/CP hybridization; Fig. 4a). Results show that, in general, capture probes with positive *n* values gave higher yields than the ones with negative ones, possibly because protruding bases provide better protein access for the crosslinker (*e.g.*: similar to a long and flexible linker). *n* = 0 appeared to be optimal in most cases (Fig. 4b).

Collectively, these results have demonstrated that the effectiveness of probes with directly conjugated crosslinkers indeed depends on the specific probe structure and the specific protein–DNA interaction, while the dual-probe strategy is more generally applicable, and it has the advantage of having a separate, tuneable, and target-binding independent probe that can effectively capture and label the protein target.

**Fig. 5:**
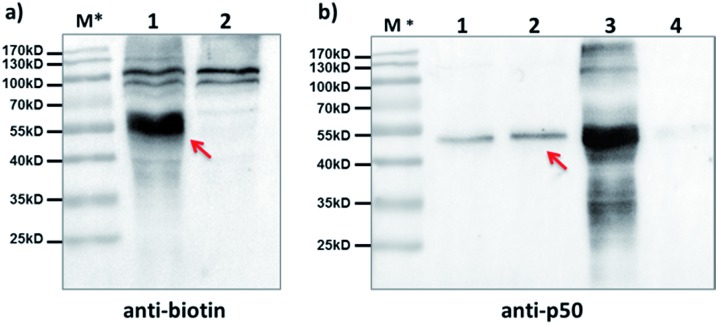
After affinity pulldown with the p50-BP and 5′-biotin-CP in cell lysate, proteins captured by streptavidin beads were eluted and blotted by (a) anti-biotin antibody; lane 1: pulldown with p50-BP/5′-biotin-CP; lane 2: pulldown with a CREB1-binding control BP/5′-biotin-CP; and by (b) anti-p50 antibody; lane 1: 0.1 pmol purified p50; lane 2: pulldown with p50-BP/5′-biotin-CP; lane 3: cell lysate only; lane 4: pulldown with a CREB1-binding BP/5′-biotin-CP. BP and CP: 2 μM each; HEK293T cell lysate: 6.8 mg mL^–1^, 1.2 mL used in pulldown; *hv*: 365 nm, 15 min, 0 °C. Elution buffer: 95% formamide, 40 mM NaOAc, 1 mM free biotin. Red arrows indicate captured p50-CP conjugates. M*: overlaid ladder.

Furthermore, we tested our method with endogenously expressed proteins. Taking advantage of the method's modularity, we used a 5′-biotin-tagged capture probe to pair with the existing p50-BP so that any p50-BP-binding proteins can be isolated by affinity pull-down. After incubation of these probes in p50-overexpressed HEK293T cell lysate, light irradiation at 365 nm, and then ultracentrifugation to remove free probes (MWCO: 50 kDa), the biotinylated species were captured by streptavidin beads. After elution, Western blots with anti-biotin and anti-p50 antibodies show protein bands matching the expected molecular weight of the p50-CP conjugate (Fig. 5a, lane 1; Fig. 5b, lane 2), which was not observed with a non-p50-binding negative control probe. These probes have shown excellent capture specificity in cell lysate with no significant enrichment of other proteins observed; a few protein bands appeared at high molecular weight in the anti-biotin blot, which may be from endogenous biotinylated species as they also showed up with the negative control (Fig. 5a, lane 2).

**Fig. 6:**
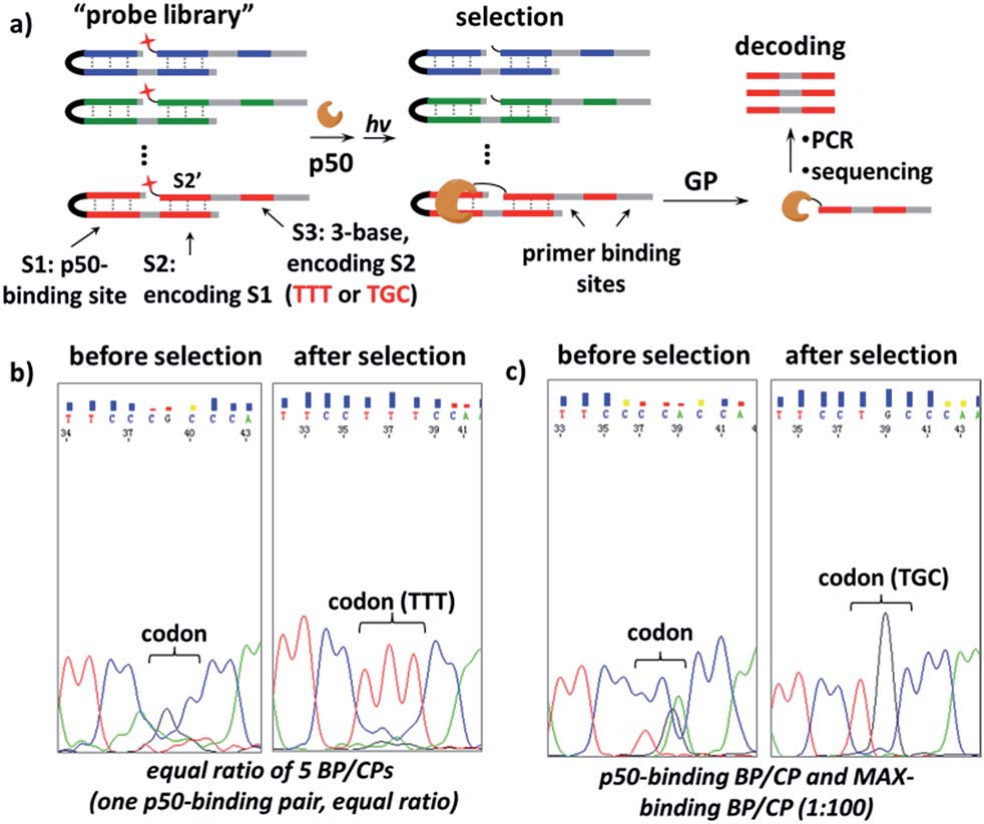
(a) Scheme for the selection of a “probe library” against the p50 target. GP: gel purification. (b) and (c) Sequencing results of the encoding S3 sites before and after selection with the probe library containing (b) equal ratio of 5 different BP/CP pairs or (c) one pair of p50-binding BP/CP and one MAX-binding BP/CP at the ratio of 1 : 100. Probe library: 20 μM; p50 target: 4 μM. Other conditions are the same as in Fig. 2. See Fig. S8 and S9† for details on the DNA sequences, selection and DNA sequencing; see Fig. S10 and S11† for full images of the sequencing data.

Further, we investigated whether our strategy can be used conversely to select protein-binding sequences from a “DNA-encoded probe library” for a particular transcription factor target, conceptually similar to the selection of DNA-encoded small molecule libraries against protein targets.^56,72–82^ Our design is shown in Fig. 6a, a “DNA-encoded probe library” contains many BP/CP pairs with different sequences. The DNA sequence of the TF-binding site (S1) in BP is encoded by the DNA sequence of the CP-hybridization site (S2). Correspondingly, the hybridization site in the complementary CP (S2′) is further encoded by a 3-base sequence (S3) at a distal location. In a library selection, the transcription factor target binds to the BP which contains matching S1 sequence, then BP templates target photo-crosslinking with the complementary CP to form the protein–CP conjugate. Therefore, the original target-binding S1 sequence can be decoded by reading the base sequence in the S3 site. In order to demonstrate this, first, a “probe library” composing of five equal ratio BP/CP pairs was prepared; in this library, only one BP/CP pair contains the matching p50-binding site, which is encoded by a “TTT” sequence in the S3 site (see details in Fig. S8†). This probe library was incubated with p50 and irradiated at 365 nm; the p50-CP conjugate generated was gel-purified, PCR-amplified and then sequenced. Results show that the p50-binding-encoding “TTT” was clearly enriched at the S3 site after selection (Fig. 6b). In a second “probe library”, a pair of p50-binding BP/CP, encoded by a “TGC” sequence at the S3 site, was mixed with 100-fold excess of MAX-binding BP/CP (see details in Fig. S9†). This library was also selected against the p50 target and again the encoding “TGC” was distinctly enriched (see the ESI for details; Fig. S10 and S11†). These selection results suggest that our strategy may be used as a selection method for the identification of target sequences for DNA-binding proteins.

**Fig. 7:**
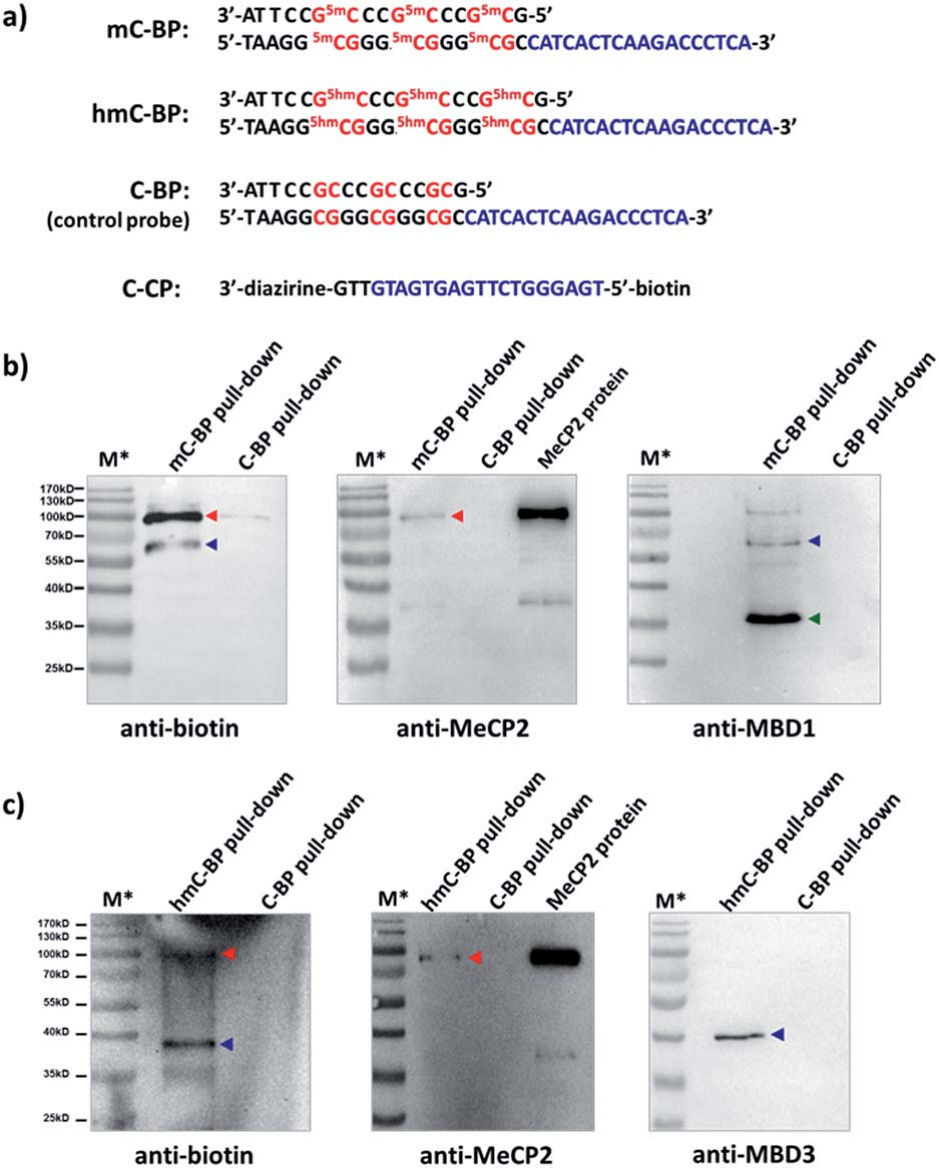
(a) Sequences of mC-BP, hmC-BP, control C-BP, and the capture probe (C-CP). ^5m^C: 5-methyl-C; ^5hm^C: 5-hydroxymethyl-C. After affinity pulldown of DNA-binding proteins in HEK293T lysate with different probes, proteins captured by streptavidin beads were eluted and blotted by different antibodies. (b) mC-BP experiments in MeCP2-overexpressed lysate. (c) hmC-BP in MeCP2-overexpressed lysate. BP and CP: 20 μM each; lysate: 7.44 mg mL^–1^, 0.2 mL used. Specific probes and antibodies used are marked. Arrows indicate captured proteins; in (b), red: MeCP2; blue: MBD1; green: possible an MBD1 degradation fragment as it did not show in the biotin blot (see Fig. S9b†); in (c), red: MeCP2; blue: MBD3. For all experiments: *hv*: 365 nm, 15 min, 0 °C; elution buffer: 95% formamide, 40 mM NaOAc, 1 mM free biotin. M*: overlaid ladder. See the ESI† for experimental details.

Finally, we studied proteins recognizing DNAs containing 5-methyl-C (mC) and 5-hydroxymethyl-C (hmC) sequences, two important epigenetic marks implicated in gene transcriptions.^83,84^ We prepared binding probes containing mC and hmC sites (mC-BP and hmC-BP; Fig. 7a), respectively, and a control probe without cytosine modification (C-BP).^85^ With the capture probe (C-CP), these probes were applied to pull-down experiments in HEK293T lysate overexpressing MeCP2, a well-known protein recognizing both of these two modifications.^85,86^ For mC-BP, Western blots showed specific enrichment of the MeCP2 protein (Fig. 7b, left and middle panels). Importantly, it was not observed with the control probe C-BP. mC-BP also specifically enriched another band at ∼65 kD, which can be blotted by the anti-MBD1 antibody, and MBD1 is known to bind mC sites on DNA.^87,88^ Similarly, for hmC-BP, specific enrichment of MeCP2 was also observed (Fig. 7c). The band at ∼40 kD was identified as possibly to be MBD3, another protein reported that is able to bind hmC.^86,89^ In addition, pull-down experiments in lysates without protein overexpression have identified several other mC- and hmC-binding proteins (Fig. S9 and S10†). Collectively, these results have demonstrated that our method may also be extended to study 5-methyl-C and 5-hydroxymethyl-C-binding proteins in epigenetic studies.

## Conclusions

In summary, we have developed a dual-probe method for characterizing transcription factor-DNA interactions and proteins recognizing epigenetic marks. By separating target recognition and capture, affinity probes can be feasibly designed to specifically capture and label DNA-binding proteins without affecting the original protein–DNA interactions. Binding probes are completely native DNAs which can be rapidly prepared in large quantity by automated DNA synthesis, making this method potentially suitable for high throughput identification of DNA-binding proteins in genomic studies.^90,91^ On the other hand, chip-based large-scale *de novo* DNA synthesis^92^ could be used to prepare probe libraries with diverse sequences, suitable for selections to identify DNA binding sequences for transcription factors and other DNA-binding proteins. Currently our laboratory is actively exploring these opportunities.
