# Supplementary material for: Photoaffinity labeling of transcription factors by DNA-templated crosslinking
Source: Chem Sci. 2014 Oct 1;6(1):745–51. doi: 10.1039/c4sc01953a (PMC5494549; doi:10.1039/c4sc01953a)
Supplement: Supplementary file 1 [file SC-006-C4SC01953A-s001.pdf]

Electronic Supplementary Material (ESI) for Chemical Science.
This journal is © The Royal Society of Chemistry 2014

Supporting Information

Photoaffinity Labeling of Transcription Factors by DNA-Templated Crosslinking

Ying Liu,[†] Wenlu Zheng,[‡] Wan Zhang,[‡] Nan Chen,[†] Yang Liu,[†] Li Chen,[†] Xiaozhou Zhou,[†] Xingshuo Chen,[†] Haifeng Zheng[†] and Xiaoyu Li[†‡]*

[†]*Key Laboratory of Bioorganic Chemistry and Molecular Engineering of the Ministry of Education, Beijing National Laboratory of Molecular Sciences (BNLMS), College of Chemistry and Molecular Engineering, Peking University, 202 Chengfu Road, Beijing, China 100871*

[‡]*Key Laboratory of Chemical Genomics, School of Chemical Biology and Biotechnology, Peking University Shenzhen Graduate School, Shenzhen, China 518055*

E-mail: xiaoyuli@pku.edu.cn

Table of Contents

1. Abbreviations. S3
2. Materials and General Methods. S5
3. Cloning, Expression and Purification of Recombinant His_6-p50 .... S6
4. Probe Structures and Oligonucleotide Synthesis, Purification, and Characterization. S7
5. General Conditions for the DNA-templated Protein Capture by Photo-crosslinking. S11
6. p50 Over-expression and Pull-down Experiment. S12
7. MeCP2 Over-expression and Pull-down Experiment. S13
8. DNA Sequences. S13
9. Oligonucleotide Characterization. S17
10. p50 Capture Mediated by Biotin-labeled CP S20
11. Comparison of p50 Captures with Different Probes in Other Types of Backgrounds. S21
12. Specificity of TBP-BP, MAX-BP and CREB1-BP in TF Protein Captures S22
13. mC-BP Pull-down Experiments in Cell Lysate without Protein Overexpression S23
14. hmC-BP Pull-down Experiments in Cell Lysate without Protein Overexpression. … S24
15. Probe Sequences Used in TF-Binding DNA Sequence Selections S25
16. Full Images of the Sequencing Results S28

1. Abbreviations.

AMA: 1:1 (v:v) aqueous methylamine (40% wt.) : aqueous ammonium hydroxide (30% wt.)

BP: binding probe

CP: capture probe

CPG: controlled-pore glass

DCC: *N*, *N*'-dicyclohexyl carbodiimide

DCM: dichloromethane

DIPEA: *N*, *N*'-diisopropylethylamine

DMEM: Dulbecco's modified eagle medium

DMF: *N*, *N*'-dimethyl formamide

DTT: dithiothreitol

DZ: diazirine

E. coil: Eschericia coli

EDC: 1-ethyl-3-(3-dimethylaminopropyl) carbodiimide hydrochloride

FAM: carboxyfluorescein

FBS: fetal bovine serum

Fmoc: 9-fluorenylmethyloxycarbonyl

FPLC: fast protein liquid chromatography

HBTU: *O*-benzotriazol-1-yl-*N*, *N*, *N*', *N*'-tetramethyluroniumhexafluorophosphate

HOBt: *N*-hydroxylbenzotriazole

IPTG: isopropyl-β-D-thiogalactosidase

LB medium: Luria-Bertani medium

NHS: *N*-hydroxysuccinimide

PBS: phosphate-buffered saline

PAGE: polyacrylamide gel electrophoresis

PMSF: phenylmethylsulphonyl fluoride

SE: succinimidyl ester

TBE: tris-borate-EDTA

TBP: TATA binding protein

TCA: trichloroacetic acid

TEAA: triethylammonium acetate

TFA: trifluoroacetic acid

2. Materials and General Methods.

Unless otherwise noted, all reagents and solvents were purchased from commercial sources and used as received. All chemical reagents were purchased from J&K Scientific Ltd. and Beijing Ouhe Technology Co. Ltd. All PCR primers and oligonucleotides with 5-methyl-C modifications were purchased from Sangon Biotech. PCR Supermix was purchased from TransGen Biotech Co. Ltd. Recombinant p50 protein was expressed in *E. coli* as the full-length human protein with an *N*-terminal polyhistidine tag as described in Section 3. Recombinant human MAX and CREB1 proteins were purchased from Sino Biological. Recombinant TBP was purchased from Active Motif and used after dialysis. p50 (12540) and MeCP2 (3456) antibodies were purchased from Cell Signaling Technology, Inc., MBD1 (ab108510), MBD2 (ab109260) and MBD3 (A2251) antibodies were purchased from Abacam. Nuclear and cytoplasmic protein extraction kit was purchased from Sangon Biotech. Water was purified with a Thermo Scientific Barnstead Nanopure system. Oligonucleotides were synthesized on standard CPG (Controlled Pore Glass, 1000 Å) beads by an automated Applied Biosystems 394 synthesizer following the machine's built-in synthesis programs. Standard phosphoramidites, other synthesis reagents and solvents were purchased from Hai Phoenix Technology and Glen Research. Anhydrous acetonitrile was freshly distilled over P_2O_5 prior to use. DNA oligonucleotides were purified by reverse-phase HPLC (Agilent 1200) using a gradient of acetonitrile (5-80%) in 100 mM TEAA (pH 7.0). Proteins and protein-DNA conjugates were analyzed by SDS-PAGE (12 % or 13.5%). DNA oligonucleotides were analyzed and purified by denaturing TBE-Urea PAGE containing 25% formamide or agarose gel electrophoresis, stained with ethidium bromide. All gel images were captured by a Bio-Rad Chemidoc system or a Tanon-1600 gel image system. Photo-crosslinking experiments were conducted by a UVP CL−1000L Ultraviolet crosslinker at 365 nm wavelengths with an intensity of approximately 100 $\mu J/cm^2$. Western blottings were performed by electro-blotting the proteins onto a PVDF membrane (Bio-Rad) at 220 mA for 1.5 hours followed by typical blocking, washing and visualization procedure.

3. Cloning, Expression and Purification of Recombinant His_6-p50.

Forward (5’-CCA GAA TTC GAG ATT ATG GCA GAA GAT GAT-3’) and reverse (5’-CCT GTC GAC ATT TTA TCC ATG CTT CAT CCC-3’) primers used in PCR were designed with plasmid DNA (pMDTM18-T-NF-*κ*B p50 vector, a gift from Prof. Huanran Tan’s laboratory at the Peking University Health Science Center), which contains the NF-*κ*B p50 gene. The restriction endonuclease sites of *EcoR*I and *Sal*I (directed by the recombinant plasmid pET-28a (+)) were introduced into the PCR product, resulting in the constructed vector of pET-28a (+)-NF-*κ*B p50 by restriction enzyme digestion and T4 ligase-mediated ligation.

BL21 (DE3) *Eschericia coli* (*E. coil*) was transformed with pET-28a (+)-NF-κB p50 and was grown in the Luria-Bertani medium containing 50 μg/mL kanamycin at 37 °C with agitation. After 1 L culture was grown to OD_{600} of 0.7-1.0, IPTG (isopropyl-*β*-D-thiogalactosidase) was then added to a final concentration of 0.8 mM. The culture was incubated at 16 °C at 200 *r*/min overnight and the cells were harvested at 4000 *rpm* for 1 hour at 4 °C. Pellets were then frozen in liquid nitrogen and stored at -80 °C. Cell pellets from the 1 L culture were re-suspended in lysis buffer (50 mM Tris-HCl pH = 7.5, 1 M NaCl, 10 mM imidazole, 1 mM PMSF, and 1 mM DTT) to a final volume of 40 mL (for 5 g of cells) and then sonicated over ice. The fusion protein was purified on a Ni-NTA resin affinity column with FPLC according to the manufacturer’s instructions (GE Healthcare; equilibrating buffer: 50 mM Tris-HCl pH = 7.5, 1 M NaCl, 1 mM imidazole, 1 mM DTT; wash buffer: 50 mM Tris-HCl pH = 7.5, 1 M NaCl, 500 mM imidazole, 1 mM DTT), which was followed by a buffer exchange with the 10K ultra-15 centrifugal filter device (Millipore) and cation exchange chromatography using HR 5/5 Mono-S (1 mL) column (GE Healthcare) with a linear gradient from 0 mM NaCl to 1 M NaCl in 10 mM Tris-HCl pH = 8.0 and 1 mM DTT. The obtained fusion protein was >95% pure as determined by SDS-PAGE analysis and it was concentrated with the 10K ultra-15 centrifugal filter device. Finally, the purified recombinant His_6-NF-*κ*B p50 protein was quantified using the Bradford assay and stored with 30% glycerol at -80 °C.

4. Probe Structures and Oligonucleotide Synthesis, Purification, and Characterization.

(a) Chemical structures of DNA-based probes.

- **Binding probe (BP):** TF-binding sequences were directly incorporated into respective binding probes via automated DNA synthesis. All BPs are native DNAs and do not have any unnatural modifications.

- **Capture probe (CP):** Diazirine was conjugated to the 3'-amino group and the "tag" (FAM or biotin) was conjugated to the 5'-amino group on modified oligonucleotides as described in detail below in **(c)**. Amine-modified oligonucleotides were prepared with the standard 5'-amino-modifier 5 and/or with the 3'-amino-modifier C7 CPG solid support. A representative chemical structure of the capture probe is shown below:

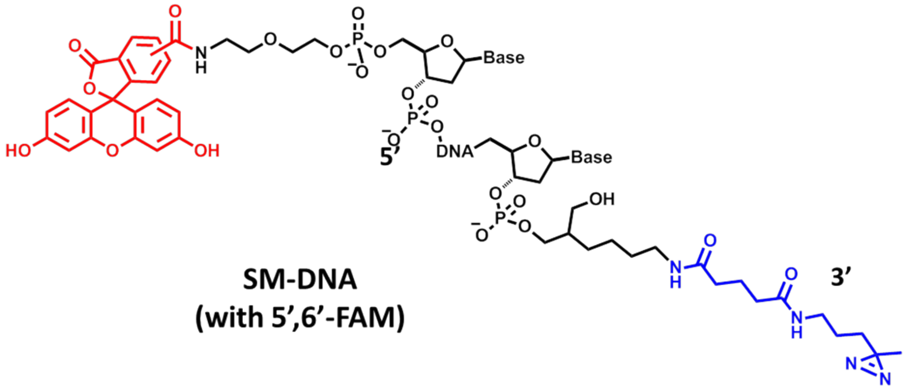

Figure S1: Representative structure of the capture probe (CP).

- **Capture probes with directly conjugated diazirine group:** diazirine was conjugated to the amino group at the 5- position of thymine bases. Amine-modified oligonucleotides were prepared with the amino modifier dT-phosphoramidite (Wuhu Huaren; see Figure S5). A representative chemical structure of the capture probe is shown below:

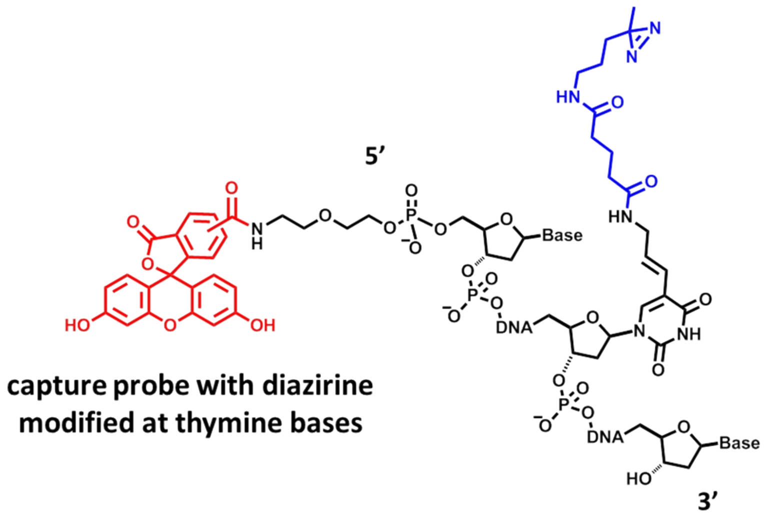

Figure S2: Representative structure of the capture probe with directly conjugated diazirine.

(b) Oligonucleotide synthesis, purification, and characterization.

DNA oligonucleotides were synthesized on an Applied Biosystems 394 DNA synthesizer using standard phosphoramidite protocols and purified by C18 reverse-phase HPLC with aqueous 0.1 M TEAA/CH_3CN gradient on Agilent 1200 HPLC systems (Eclipse-XDB C18, 5 µM, 9.4 x 200 mm or 4.6 x 150 mm). After solid-phase synthesis, oligonucleotides on CPGs were cleaved by AMA (55ºC, 55 min) over a dry bath, then concentrated, neutralized (2.0 M TEAA) before HPLC purification. For all non-standard phosphoramidites (other than standard dA-, dT-, dC- and dG-phosphoramidites), coupling time was modified to 999 seconds. Non-standard phosphoramidites were purchased from Glen Research and Wuhu Huaren. Oligonucleotides were quantitated by a BioTek Epoch UV-Vis spectrometer based on extinction coefficient at 260 nm. Oligonucleotides were characterized by either a Bruker APEX IV (for ESI-MS) or a Bruker ultrafleXtreme [for MALDI-MS, matrix: 8:1 (50 mg/mL 3-HPA in 1:1 water: acetonitrile): (50 mg/mL ammonium citrate in water)] mass spectrometer. All DNA sequences are written in 5'- to 3'- orientation unless otherwise noted.

Oligonucleotides with 5-hydroxymethyl-C modifications were prepared on an ABI Expedite 8909 synthesizer using synthesis and purification protocols as recommended by Glen Research. Briefly, after solid-phase synthesis, CPG was add with 1 mL of 0.4 M NaOH in MeOH/water 4:1 (v/v) and the cleavage suspension was maintained at room temperature for 17 hours. The cleavage solution was diluted to 10 mL with 100 mg/mL NaCl in water and then loaded onto a pre-prepared Glen-Pak DNA cartridge (60-5200) fitted with a 10 mL syringe. The oligonucleotide solution was purified with the standard protocol except an additional salt wash step at the beginning.

(c) Oligonucleotide modification and probe synthesis.

(i) Binding probes.

All binding probes are native DNAs and were synthesized, purified and characterized as described in the general methods.

(ii) Dual-labeled capture probes with FAM and diazirine groups.

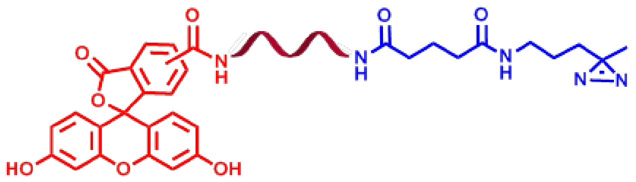

DNA synthesis was performed with the 3'-amino-modified CPG. MMT was deprotected with 3% TCA. 5, 6-FAM (15.0 mg, 40 µmol) was dissolved in 200 µL anhydrous DMF with 1 equiv. HBTU (15.2 mg, 40 µmol). After 1 hour at room temperature, the activation mixture was added to the CPG along with 2.3 equiv. DIPEA (15.2 µL, 92 µmol). The suspension was agitated at 37 ºC overnight with vortex. The CPG was washed with DMF (3x 600µL), CH_3CN (3x 600µL), and dried by gentle air stream. The FAM-labeled DNA was cleaved by AMA, desalted by a NAP-10 column (GE Pharmacia), and purified by HPLC.

Bis(2, 5-dioxopyrrolidin-1-yl) glutarate (uncleavable linker, 0.7 mg, 2.1 µmol) and 3-(3-methyl-3H-diazirin-3-yl) propan-1-amine (diazirine, 0.8 µL, 5.6 µmol) were dissolved in 20 µL anhydrous DMSO respectively. The uncleavable linker solution was first added to the FAM-labeled DNA (in 80 µL phosphate buffer, 0.25 M, pH 7.2) with sonication at 40 ºC. After 2.5 minutes the diazirine solution was added to the mixture. The reaction was maintained at 40 ºC for 1 hour and then desalted by NAP-5 (GE Pharmacia). The final product was purified by HPLC.

(iii) Dual-labeled capture probes with biotin and diazirine groups.

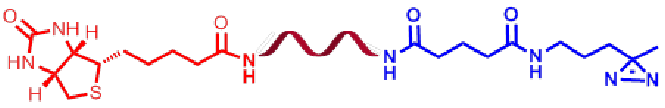

The synthesis procedure was the same as described in **(ii)** except that biotin carboxylic acid was used instead of the 5, 6-FAM carboxylic acid.

(iv) Probes with directly conjugated diazirine.

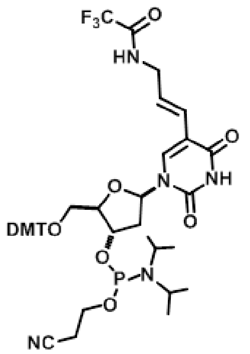

Figure S3: Structure of the amino-modifier dT-phosphoramidite used in probe synthesis.

DNAs were synthesized using the amino-modifier dT-phosphoramidite (Figure S5; Wuhu Huaren) to replace the original nucleobase dT in DNA sequences. The TFA protecting group was removed during AMA cleavage to expose the amino group, which was conjugated with the diazirine as described in (**iii**) to afford the backbone-modified DNA (Figure S2).

5. General Conditions for the DNA-templated Protein Capture by Photo-crosslinking.

Typically each reaction mixture contains 2 μM of proteins and 2 μM of each probes in a binding buffer (10 mM $MgCl_2$, 50 mM KCl, 10 mM EDTA, 25m M DTT, and 1x PBS). The final reaction volume is 10 μL. For experiment with cell lysate background, cells were lysed with standard RIPA buffer and the total protein concentration was measured by the Bradford assay. Protein concentrations were calculated based on protein's absorbance at 280 nm and known extinction coefficients. In general, BP was first heated to 95 ºC briefly and then cooled down to room temperature slowly to form the hairpin structure before protein and/or lysate was added. The mixture was incubated at 37 ºC for 30 minutes before CP addition. The reaction mixture was then irradiated at 365 nm for 15 minutes at 0 °C for crosslinking. The irradiated sample was heated at 95 ºC for 5 minutes and then analyzed by SDS-PAGE; the gel was visualized and quantitated by UV trans-illumination and densitometry.

For all experiments monitored by FAM fluorescence, a filter at 365 nm was used for detection and quantitation. For affinity pull-down experiments with the biotin-labeled CP to enrich captured

proteins, we first pretreated the cell lysate (NF-κB p50 over-expressed 293T) with immobilized streptavidin agarose beads (50-100 μL) at 4 ºC overnight in order to remove any endogenous biotinylated species. Pretreated cell lysate was added with 1/10 volume of the binding buffer (10x) and $MgCl_2$ (100 mM), which was then subjected to the experiment conditions described above. The mixture was filtered to remove excess free DNA probes with an ultra-filtration tube (Millipore) at 4 ºC. To thoroughly remove free probes, the filtration pellet was usually washed with 1x PBS (10x 300 μL). The washed pellet was collected and washed again with 50 μL 1x PBS before being added to a slurry of streptavidin agarose beads (150 μL). After incubation for 2 hours, the beads were washed with 1x PBS (3x 300 μL), 1x PBS/0.5 M NaCl (3x 300μL), RIPA buffer (3x 300 μL), 1x PBS/Triton X100/0.1% SDS (3x 300 μL), and 1x PBS (3x 300 μL) to thoroughly remove any non-covalently-bound proteins. An elution solution with 95 % formamide, 40 mM NaOAc, and 1 mM free biotin (80 μL) was used to elute enriched proteins before analysis.

6. p50 Over-expression and Pull-down Experiment.

HEK 293T cells were cultured in DMEM medium with 10% FBS until 70% coverage, then transfected with the NF-κB p50 plasmid with lipofectine 2000 (Invitrogen) for 2 days. The transfected HEK 293T cells were then lysed by standard RIPA buffer and the total protein concentration was measured to be 6.80 mg/mL by Bradford assay. p50 over-expression was confirmed by Western blot (Figure S6). 1200 μL of cell lysate was subjected to the protein capture and pull-down experiments as described above in Section 5.

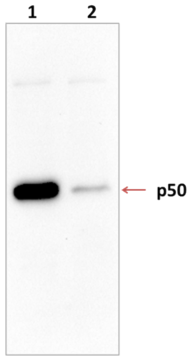

Figure S4: p50 overexpression in transfected HEK293T cells, monitored by p50-specific antibody. Lane 1: transfected cells; lane 2: control HEK293T cells.

7. MeCP2 Over-expression and Pull-down Experiment.

HEK 293T cells were cultured in DMEM medium with 10% FBS until 70% coverage, then transfected with the MeCP2 plasmid with lipofectine 2000 (Invitrogen) for 2 days. The transfected HEK 293T cells were then lysed by a nuclear and cytoplasmic protein extraction kit and the total protein concentration was measured to be 7.44 mg/mL by Bradford assay. MeCP2 over-expression was confirmed by Western blot (Figure S5). 200 µL of nuclear extract was subjected to the protein capture and pull-down experiments as described above in Section 5.

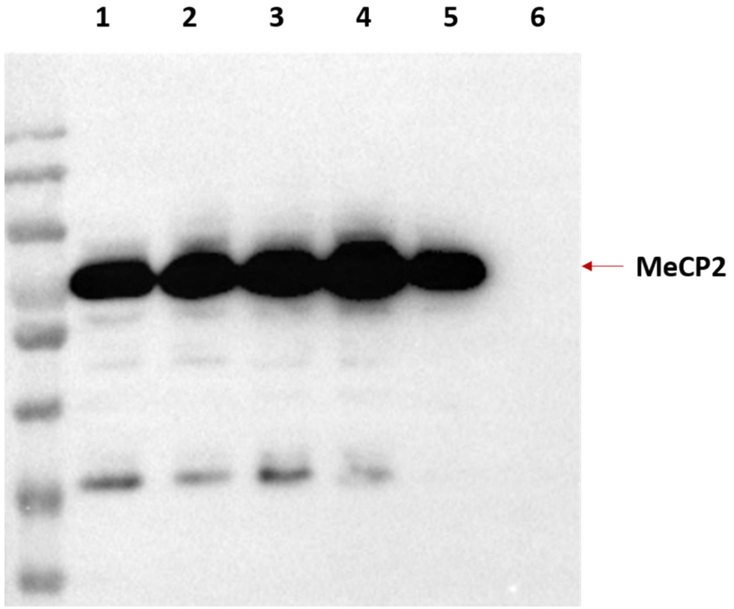

Figure S5: MeCP2 overexpression in transfected HEK293T cells, monitored by MeCP2-specific antibody. Lane 1-5: transfected cells; lane 6: control HEK293T cells.

8. DNA Sequences.

- **Binding probes and capture probes (Figure 2 & 3):**

Binding probe (p50-BP): 5’-G***GGAAATTCCC***GTTTC***GGGAATTTCC***CCACTCAAGACCCTCACAAACGCCC-3’

Binding probe (MAX-BP): 5’-G***CACGTGG***GTTTC***CCACGTG***CCACTCAAGACCCTCACAAACGCCC-3’

Binding probe (TBP-BP): 5'-G***TTTTATA***GTTTC***TATAAAA***CCACTCAAGACCC TCACAAACGCCC-3'

Binding probe (CREB1-BP): 5'-G***TGACGTCA***GTTTC***TGACGTCA***CCACTCAAGACCC TCACAAACGCCC-3'

Capture probe (p50): 5'-TGAGGGTCTTGAGTG-3'

Capture probe (MAX): 5'-TTGTGAGGGTCTTGA-3'

Capture probe (TBP): 5'-TGAGGGTCTTGAGTG-3'

Capture probe (CREB1): 5'-TGAGGGTCTTGAGTG-3'

Capture probe (mismatched, for p50, MAX, and CREB1): 5'-GATCTCCCATTGCTT-3'

Capture probe (mismatched, for TBP): 5'-GATCTCCCATTGCTT-3'

- **Probes with directly conjugated diazirine (Figure 3):**

p50-T1: 5'-TCA***GGGAATTTCC***TCA-3'

p50-T2: 5'-TCA***GGGAATTTCC***TCA-3'

p50-T3: 5'-TCA***GGGAATTTCC***TCA-3'

p50-T4: 5'-GGGTCTTGAGTAG***GGAAATTCCC***GTTTC***GGGAATTTCC***CTACTCA-3'

Complementary DNA sequence: 5'-TGA***GGAAATTCCC***TGA-3'

TBP-T1: 5'-TCA***TTTTATA***TCA-3'

TBP-T2: 5'-TCA***TTTTATA***TCA-3'

TBP-T3: 5'-GGGTCTTGAGTAG***TTTTATA***GTTTC***TATAAAA***CTACTCA-3'

Complementary DNA sequence: 5'- TGA***TATAAAA***TGA-3'

MAX-T1: 5'-TCA***CCACGTG***TCA-3'

MAX-T2: 5'-TCA***CCACGTG***TCA-3'

MAX -T3: 5'-GGGTCTTGAGTAG***CACGTGG***GTTTC***CCACGTG***CTACTCA-3'

Complementary DNA sequence: 5'-TGA***CACGTGG***TGA-3'

CREB1-T1: 5'-TCA***TGACGTCA***TCA-3'

CREB1-T2: 5'-TCA***TGACGTCA***TCA-3'

CREB1-T3: 5' -TCA***TGACGTCA***TCA-3'

Complementary DNA sequence: 5'-TGA***TGACGTCA***TGA-3'

- **Probes used in the "*n*" value study (Figure 4):**

Binding probe (p50-BP): 5'-G***GGAAATTCCC***GTTTC***GGGAATTTCC***CCACTCAAGACCCTCACAAACGCCC-3'

Binding probe (MAX-BP): 5'-G***CACGTGG***GTTTC***CCACGTG***CCACTCAAGACCCTCACAAACGCCC-3'

Binding probe (TBP-BP): 5'-G***TTTTATA***GTTTC***TATAAAA***CCACTCAAGACCC TCACAAACGCCC-3'

Capture probes:

Capture probe (n = -9): 5'-GGG CGT TTG TGA GGG-3'

Capture probe (n = -6): 5'-CGT TTG TGA GGG TCT-3'

Capture probe (n = -3): 5'-TTG TGA GGG TCT TGA-3'

Capture probe (n = 0): 5'-TGA GGG TCT TGA GTG-3'

Capture probe (n = 3): 5'-TGA GGG TCT TGA GTG TTG-3'

Capture probe (n = 6): 5'-TGA GGG TCT TGA GTG TTG GGT-3'

Capture probe (n = 9): 5'-TGA GGG TCT TGA GTG TTG GGT CAG-3'

- **Endogenous p50 pull-down sequence (Figure 5):**

Binding probe (p50-BP): 5'-G***GGAAATTCCC***GTTTC***GGGAATTTCC***CCACTCAAGACCCTCACAAACGCCC-3'

Capture probe (with biotin): 5'-TGAGGGTCTTGAGTG-3'

- **Probes used in TF-binding DNA sequence selection (Figure 6):**

See Figure S13-S14 for details.

- **Multiplexed protein capture probes (Figure S12):**

Binding probe (p50-BP): 5’-
G***GGAAATTCCC***GTTTC***GGGAATTTCC***CCCTACTTGCCTTAGT-3’
Binding probe (MAX-BP): 5’-
G***CACGTGG***GTTTC***CCACGTG***CCACTCAAGACCCTCACAA -3’
Binding probe (CREB1-BP):
5’-G***TGACGTCA***GTTTC***TGACGTCA***CTCGTTCGTTACC CTC-3’

Capture probe (p50-CP): 5’-ACTAAGGCAAGTAGG-3’
Capture probe (MAX-CP): 5’-TTGTGAGGGTCTTGA-3’
Capture probe (CREB1-CP): 5’-GAGGGTAACGAACGA-3’

- **DNA sequences with 5-methyl-C and 5-hydroxymethyl-C, as well as the complementary CP, are shown in Figure 7 of the main text.**

- Bold and italic letters indicate TF-binding DNA sequences; underlined letters indicate diazirine conjugation sites.

9. Oligonucleotide Characterization.

Oligonucleotide	Expected mass (Da)	Observed mass (Da)	Note
p50-BP (Figure 2)	15509.1	15516.9	MALDI
CP (Figure 2)	5620.1	5622.3	ESI
CP (mismatched for p50, MAX, CREB1)	5435.0	5433.0	ESI
p50-T1	5608.0	5630.8	ESI $(M+Na)^+$
p50-T2	5608.0	5609.2	ESI
p50-T3	5608.0	5609.2	ESI
p50-T4	14609.9	14593.6	ESI
complementary DNA to the p50-T series	4905.3	4905.6	ESI
TBP-BP (Figure 3)	13652.0	13658.1	MALDI
TBP-CP (Figure 3)	5970.3	5970.6	ESI
CP (mismatched for TBP)	5785.2	5785.6	ESI
MAX-BP (Figure 3)	13656.9	13664.8	MALDI
MAX-CP (Figure 3)	5463.1	5465.6	ESI
CREB1-BP (Figure 3)	13652.0	13660.4	MALDI
CREB1-CP (Figure 3)	5620.1	5622.3	ESI
TBP-T1	5043.2	5082.7	ESI $(M+K)^+$
TBP-T2	5043.2	5044.1	ESI
TBP-T3	13120.4	13122.6	ESI
complementary DNA to the TBP-T series	4005.7	4006.2	ESI
MAX-T1	4632.4	4635.4	ESI
MAX-T2	4632.4	4634.6	ESI
MAX-T3	12419.4	12442.6	ESI $(M+Na)^+$
complementary DNA to the MAX-T series	4014.7	4016.5	ESI
CREB1-T1	4960.6	4962.8	ESI

CREB1-T2	4960.6	4963.2	ESI
CREB1-T3	4960.6	4962.4	ESI
complementary DNA to the CREB1-T series	4302.9	4303.1	ESI
p50-BP (Figure 4)	15509.1	15516.9	MALDI
MAX-BP (Figure 4)	11857.7	11849.4	MALDI
TBP-BP (Figure 4)	13652.0	13658.1	MALDI
CP (Figure 4; n = -9)	5661.1	5661.3	ESI
CP (Figure 4; n = -6)	5571.1	5571.7	ESI
CP (Figure 4; n = -3)	5595.1	5595.2	ESI
CP (Figure 4; n = 0)	5620.1	5622.3	ESI
CP (Figure 4; n = 3)	6557.7	6558.2	ESI
CP (Figure 4; n = 6)	7520.3	7521.5	ESI
CP (Figure 4; n = 9)	8451.9	8452.6	ESI
CP (Figure 4; n = -9)	5529.1	5529.3	ESI
CP (Figure 4; n = -6)	5439.1	5439.6	ESI
CP (Figure 4; n = -3)	5463.1	5464.2	ESI
CP (Figure 4; n = 0)	5488.1	5488.4	ESI
CP (Figure 4; n = 3)	6425.7	6425.8	ESI
CP (Figure 4; n = 6)	7388.3	7388.3	ESI
CP (Figure 4; n = 9)	8319.9	8320.6	ESI
p50-BP (Figure 5)	15509.1	15516.9	ESI
biotin-CP (Figure 5)	5488.1	5488.4	ESI
p50-BP (Figure S8)	12822.4	12815.4	ESI
MAX-BP (Figure S8)	11857.7	11849.4	ESI
CREB1-BP (Figure S8)	11562.5	11569.6	ESI
p50-CP (Figure S8)	5591.1	5631.5	ESI $(M+K)^+$
MAX-CP (Figure S8)	5595.1	5597.5	ESI
CREB1-CP (Figure S8)	5616.1	5618.4	ESI

Pair #1 BP (Figure S13)	11989.8	11989.8	ESI
Pair #2 BP (Figure S13)	10181.7	10181.7	ESI
Pair #3 BP (Figure S13)	10177.6	10178.1	ESI
Pair #4 BP (Figure S13)	3728.5	3797.7	ESI $(M+3Na)^+$
Pair #5 BP (Figure S13)	8314.5	8313.5	ESI
Pair #1 CP (Figure S13)	10620.3	10693.2	ESI $(M+3Na)^+$
Pair #2 CP (Figure S13)	10590.3	10621.6	ESI $(M+Na)^+$
Pair #3 CP (Figure S13)	10559.3	10571.4	ESI
Pair #4 CP (Figure S13)	10572.3	10576.5	ESI
Pair #5 CP (Figure S13)	10597.3	10712.9	ESI $(M+3K)^+$
Pair #1 BP (Figure S14)	11989.8	11989.8	ESI
Pair #2 BP (Figure S14)	10177.6	10178.1	ESI
Pair #1 CP (Figure S14)	10630.3	10708.2	$(M+2K)^+$
Pair #2 CP (Figure S14)	10559.3	10596.0	$(M+K)^+$
mC-BP (long, Figure 7)	10464.8	10463.6	MALDI
mC-BP (short, Figure 7)	4821.1	4822.6	MALDI
mC-CP (Figure 7)	7372.3	7372.6	ESI
hmC-BP (long, Figure 7)	10512.8	10512.0	ESI
hmC-BP (short, Figure 7)	4869.1	4869.1	ESI
C-BP (long, Figure 7)	10422.8	10421.6	MALDI
C-BP2 (short, Figure 7)	4779.1	4779.0	MALDI

10. p50 Capture Mediated by Biotin-Labeled CP.

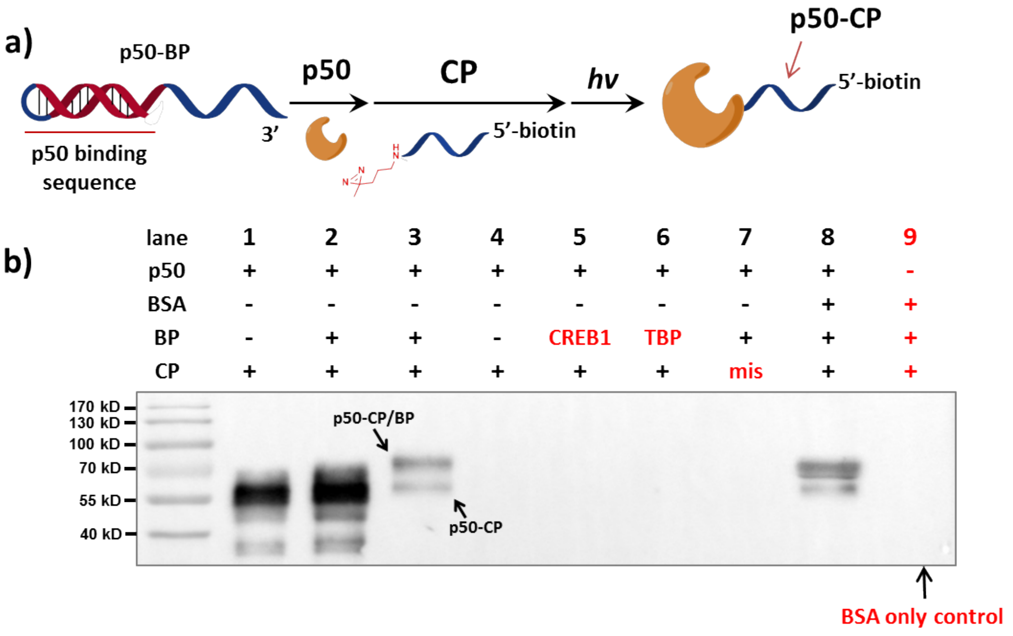

Figure S6: a) Reaction scheme of p50 labeling with p50-BP and biotin-CP. b) Reaction analyzed by anti-biotin Western blot. p50-BP, biotin-CP, p50: 2 µM each; BSA in lane 8 and 9: 20 µM. *hv*: 365 nm, 15 min, 0 °C. Buffer: 10 mM $MgCl_2$, 50 mM KCl, 10 mM EDTA, 25 mM DTT, 1x PBS. Lane 1-2: standard samples of p50-CP conjugate and p50-CP conjugate with p50-BP; lane 3: p50 labeling by p50-BP/biotin-CP with *hv*; lane 4-8: same as lane 3 but without BP, with a non-p50-binding (CREB1-binding) BP, with a non-p50-binding (TBP-binding) BP, with mismatched CP, and with additional 20 µM BSA; lane 9: the BSA only control, in which 20 µM of BSA without any p50 was subjected to the same photo-crosslinking procedure by p50-BP/biotin-CP and no BSA labeling was observed. p50-conjugated CP and BP DNAs may partially renature in gel, resulting two product bands: the p50-CP conjugate and p50-CP/BP duplex, as marked by arrows.

The above experiment is similar to the Figure 2b experiment except that 5'-biotin-CP was used instead of the 5'-FAM-CP. This has shown the modularity of our method where the same BP can be paired with different CPs bearing different tags. In addition, similar labeling specificity was observed.

11. Comparison of p50 Captures with Different Probes in Other Types of Background.

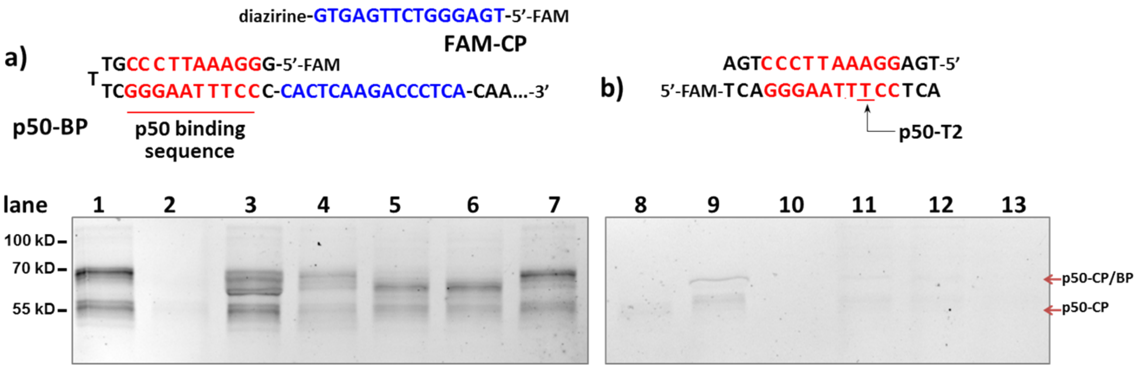

Figure S7: Comparison of p50 capture by p50-BP/CP and p50-T2 in other types of background. a) p50-BP and FAM-CP sequences and PAGE analysis result, monitored by FAM fluorescence. b) Control probe p50-T2 sequence and AGE analysis result, monitored by FAM fluorescence. Diazirine modification site is underlined and marked in b). Lane 1: p50 capture by p50-BP/CP with irradiation; lane 2: p50 with only CP after irradiation (negative control); lane 3-6: same as lane 1 but in the background of BSA, cardiomyocyte nuclear extract, HeLa cell lysate, and MCF7 cell lysate, respectively; lane 7: same as lane 4 but cardiomyocyte nuclear extract was added after irradiation to control for lysate's effect on fluorescence detection; lane 8-13: same as lane 2-7 but with p50-T2 instead of p50-BP/CP. Reaction conditions are the same as in Figure 2 of the main text. Expected positions of protein-DNA conjugates are marked by arrows.

In order to further compare the DNA-based dual probe method with probes having directly conjugated diazirine, we performed additional experiments in several other types of background: non-specific BSA, cardiomyocyte nuclear extract, HeLa lysate and MCF7 lysate (Figure S7). Results show that p50-BP/CP can efficiently capture the p50 protein in all cases, while the p50-T2 probe with modified protein-binding site was not able to efficiently capture p50 in these scenarios.

12. Specificity of TBP-BP, MAX-BP and CREB1-BP in TF Protein Captures.

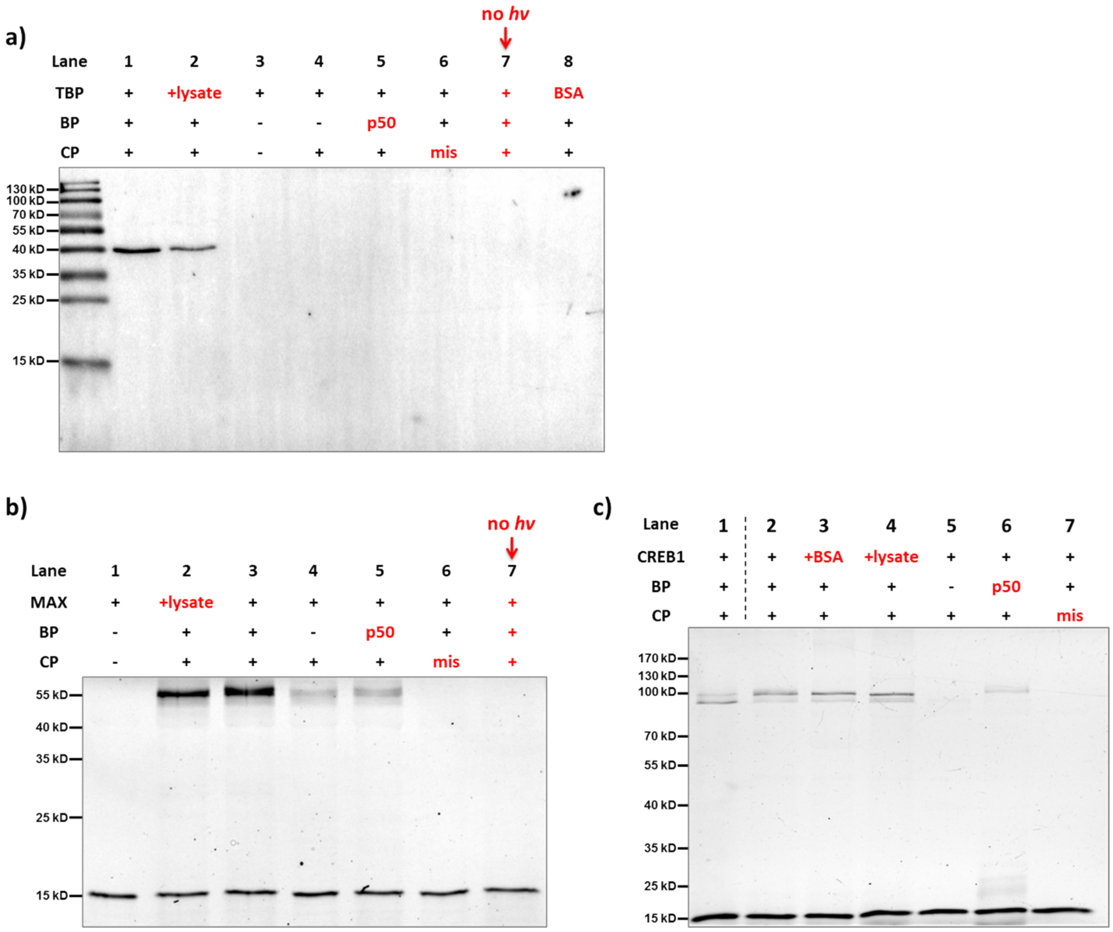

Figure S8: Capture of TBP, MAX, and CREB1 proteins by respective BP/CP probes along with other control experiments. Reactions setup and conditions are the same as in Figure 2a and 2b of the main text. a) TBP experiments. Lane 1: TBP capture by BP/CP with *hv*; lane 2: same as 1 but with added lysate; lane 3: TBP only; lane 4-8: same as 1 but without BP, with a non-TBP-binding (p50-binding) BP, with mismatched CP, without irradiation, and with 1 eq. BSA (no TBP). b) MAX experiments. Lane 1: MAX only; lane 2: TBP capture by BP/CP with *hv* with added lysate; lane 3: TBP capture by BP/CP with *hv*; lane 4-7: same as lane 3 but without BP, with a non-MAX-binding (p50-binding) BP, with mismatched CP, without irradiation. c) CREB1 experiments. Lane 1: CREB1-CP/BP standard sample; lane 2: CREB1 capture by BP/CP with *hv*; lane 3: same as lane 2 with additional 1 eq. BSA; lane 4: same as lane 2 with added lysate; lane 5-7: same as lane 2 but without BP, with a non-CREB1-binding (p50-binding) BP, with mismatched CP. Lysate: HeLa, 4.8 mg/mL.

Similar to the experiments shown in Figure 2b of the main text, we performed series of control experiments with TBP, MAX and CREB1. Results show that captures of these TF proteins also require both specific TF-DNA interaction and photo-crosslinking mediated by a complementary CP.

13. mC-BP Pull-down Experiments in Cell Lysate without Protein Overexpression.

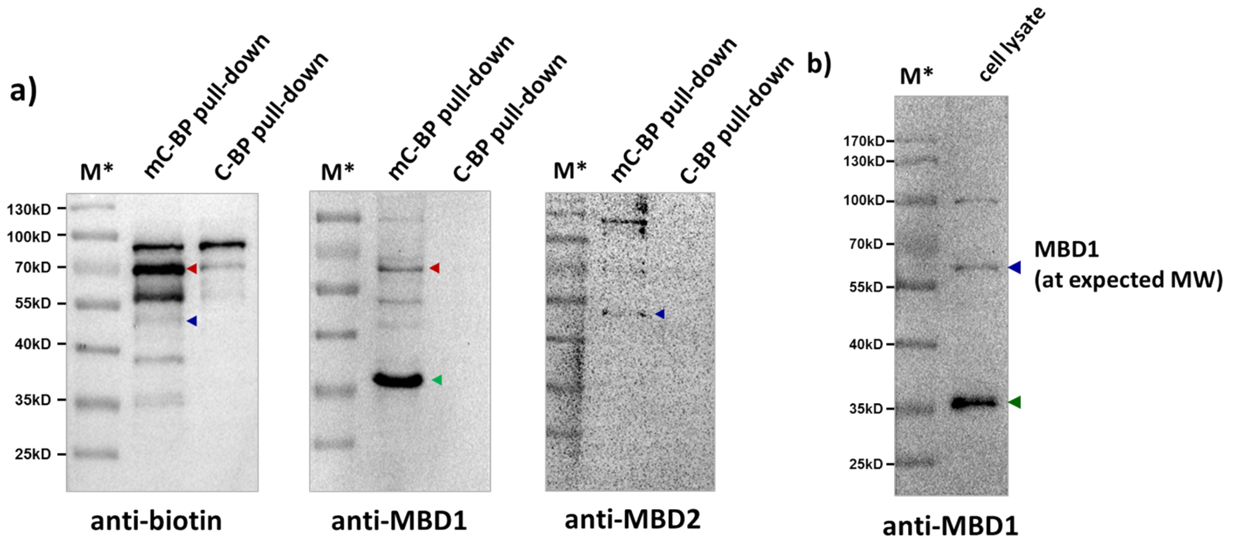

Figure S9: a) After affinity pulldown of DNA-binding proteins in HEK293T cell lysate (no protein overexpression) with different probes, proteins captured by streptavidin beads were eluted and blotted by antibodies. Sequences of mC-BP, negative control C-BP, and capture probe (C-CP) are shown in Figure 7 of the main text. Probes and antibodies used are marked. BP and CP: 20 µM each; cell lysate: 8.26 mg/mL, 0.2 mL used in pull-down. Red arrows: MBD1; blue arrows: MBD2; green arrow: possible MBD1 fragment. b) MBD1 antibody (Abcam, ab108510) blotting test in HEK293T lysate (no probes). For all experiments: *hv*: 365 nm, 15 min, 0 °C.; elution buffer: 95 % formamide, 40 mM NaOAc, 1 mM free biotin. M*: overlaid ladder.

We performed pull-down experiments in HEK293T lysate without MeCP2 over-expression with the **mC-BP** probe. Multiple protein bands were enriched by **mC-BP** specifically, in comparison with the non-methylated control probe. Two of them were identified to be possibly the MBD1 and MBD2 proteins by Western blots (red and blue arrows, Figure S9a). The band at ~55 KD matches the molecular weight of MBD4. All these MBD proteins are known to more preferably bind to 5-methyl-C-containing DNAs than the DNAs with non-methylated sequences (B. Hendrich and A. Bird *Mol. Cell Biol.* **1998**, *18*, 6538). The strong a band at ~36 KD in the MBD1 blot did not appear in the biotin blot (Figure S9a, middle panel, green arrow). The anti-MBD1 antibody we used (Abcam, catalog#: ab108510) also strongly blotted this band in HEK293T lysate alone (without any probes; Figure S9b, green arrow), therefore we hypothesize that it might be a degradation fragment of MBD1. The non-specifically enriched band ~90 KD may be endogenous cellular biotinylated species.

14. hmC-BP Pull-down Experiments in Cell Lysate without Protein Overexpression.

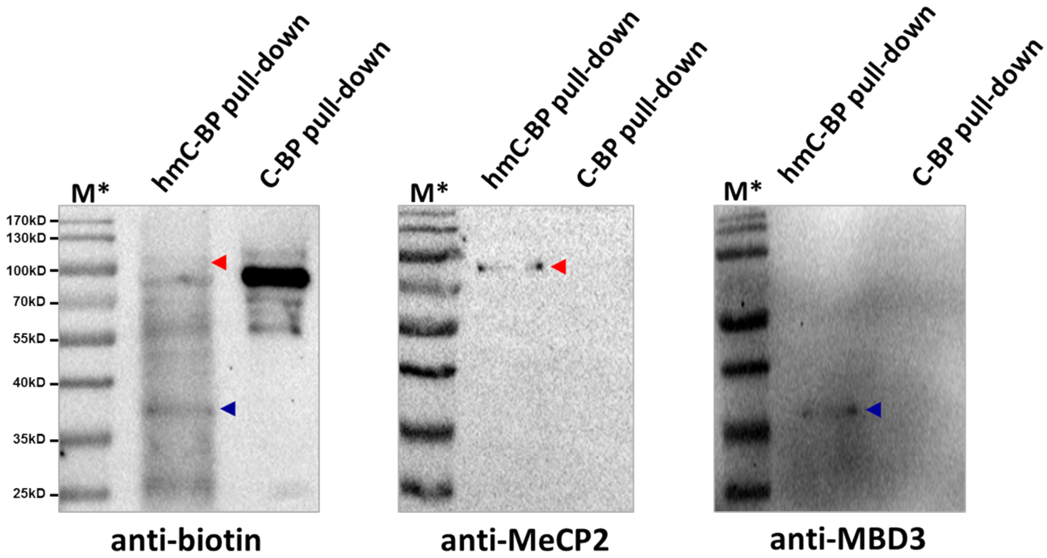

Figure S10: After affinity pulldown of DNA-binding proteins in HEK293T cell lysate (no protein overexpression) with different probes, proteins captured by streptavidin beads were eluted and blotted by antibodies. Sequences of hmC-BP, control C-BP, and capture probe (C-CP) are shown in Figure 7 of the main text. Probes and antibodies used are marked. BP and CP: 20 µM each; lysate: 7.94 mg/mL, 0.2 mL used in pull-down. Red arrows: MeCP2; blue arrows: MBD3. For all experiments: *hν*: 365 nm, 15 min, 0 °C.; elution buffer: 95 % formamide, 40 mM NaOAc, 1 mM free biotin. M*: overlaid ladder.

We performed pull-down experiments in HEK293T lysate without MeCP2 over-expression with the **hmC-BP** probe, which generated multiple and quite smeared protein bands. However, both MeCP2 and MBD3 can still be detected by Western blots (Figure S10). Importantly, they were not detected in control experiments with non-hydroxymethylated-C probes, suggesting the capture specificity. The strong band ~90 KD in the C-BP lane may be endogenous cellular biotinylated species that was not completely removed while pre-treating the lysate with streptavidin beads.

15. Probe Sequences Used in TF-Binding DNA Sequence Selections.

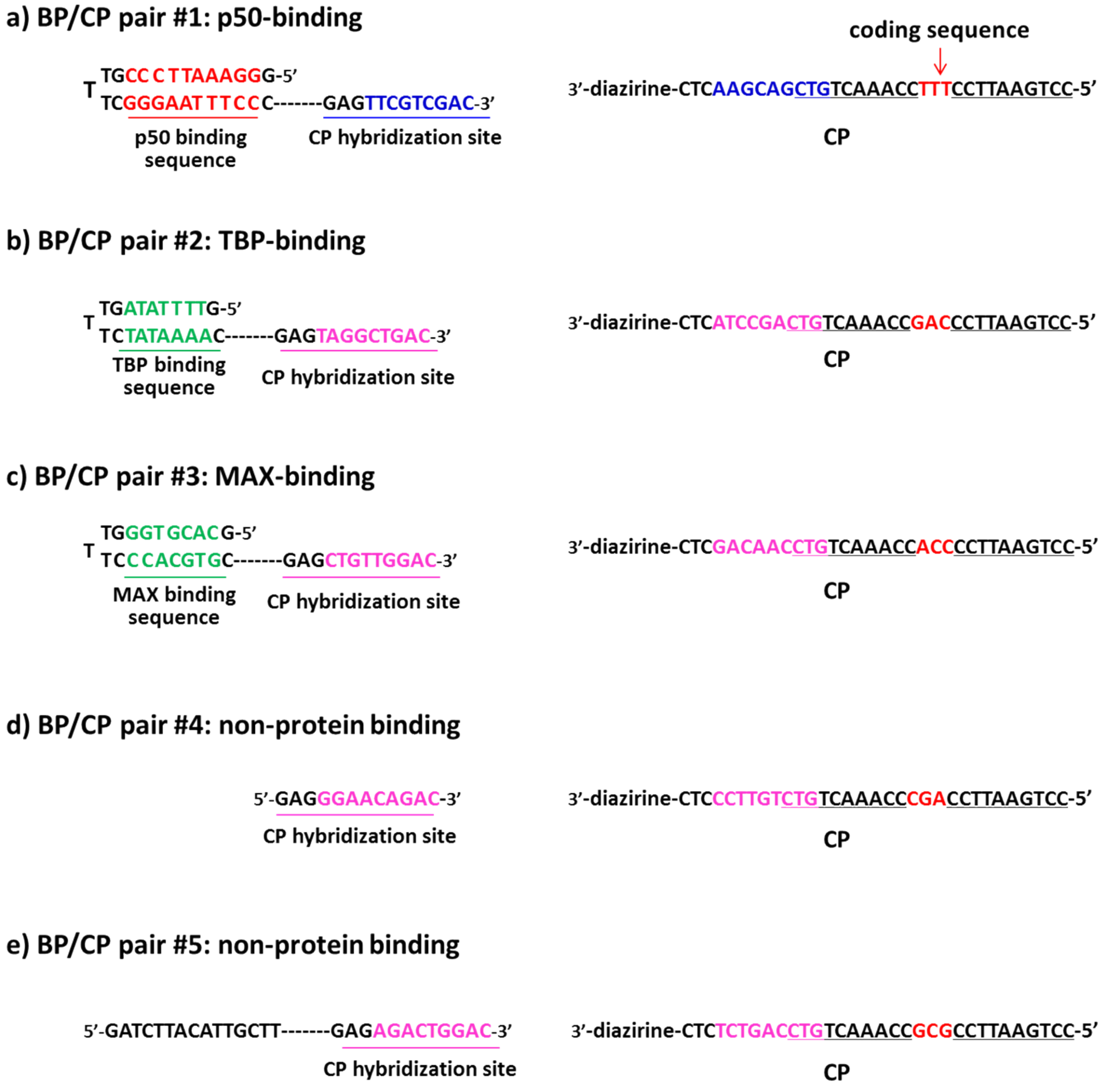

Figure S11: Complete sequences of BP/CP pairs used in TF-binding DNA sequence selection experiment (Figure 6b of the main text). a)-e): Five pairs of BP/CPs used in the equal ratio library selection. Underlined sequences in CPs: PCR primer binding sites.

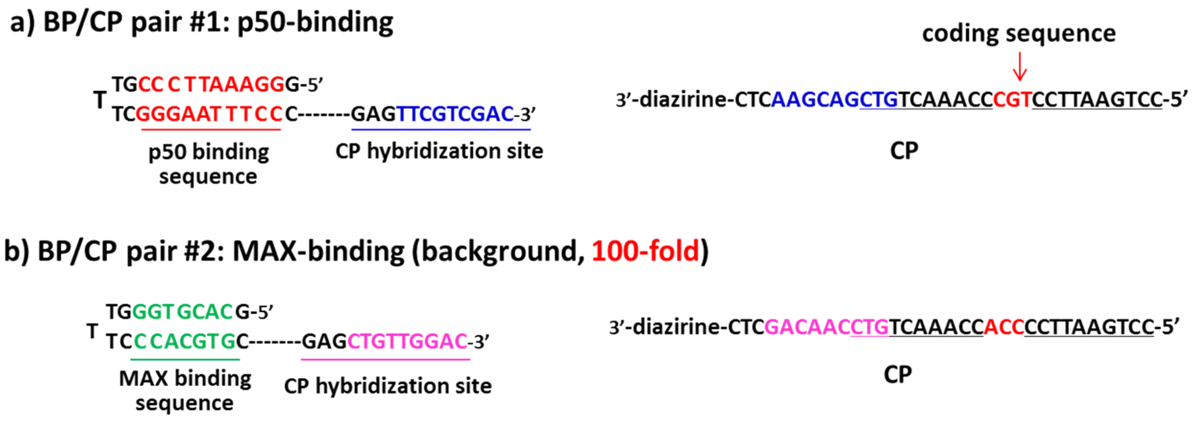

Figure S12: Complete sequences of BP/CP pairs used in p50-binding DNA sequence selection (Figure 6c of the main text). a)-b): Two pairs of BP/CPs used in the 1:100 library selection. Underlined bases in CPs: PCR primer binding sites.

Figure S11 and S12 show the detailed probe structures and sequences used in Figure 6's selection experiments. In the 1st experiment (Figure 6b), equal molar ratio of BP/CP pairs of a)-e) were mixed; in the 2nd experiment (Figure 6c), 100-fold excess of MAX-binding BP/CP was mixed with p50-binding BP/CP.

Experimentally, these probes were mixed, heated briefly to 95 °C and then cooled down slowly to room temperature. p50 was added and the mixture was incubated at 37 °C for 30 min (Probe library: 20 µM; p50 target: 4 µM). After the incubation, the mixture was irradiated under 365 nm as described above for photo-crosslinking. The mixture was then loaded onto SDS-PAGE and the protein-CP conjugate band was cut out before extraction by 1x PBS. 1/10 of the extract was used for PCR amplification. The PCR product was directly sent for sequencing (Majorbio Inc., Beijing) without further purification.

- **PCR amplification primers:**

  5'-
  TTGTGATAATATACAGGACAATGACCAGATATAGTCAAGATTTACATGCCTGACC
  TGAATTCC-3'

5'-
ACTAATCCAAGTACTCCGGAACATCAAGGACCCTATACTTTAAGTGTGTACATGA
CAGTTTGG-3'

- **Sequencing primers:**

  5'- TTGTGATAATATACAGGA-3'

  5'- ACTAATCCAAGTACTCCG-3'

16. Full Images of the Sequencing Results.

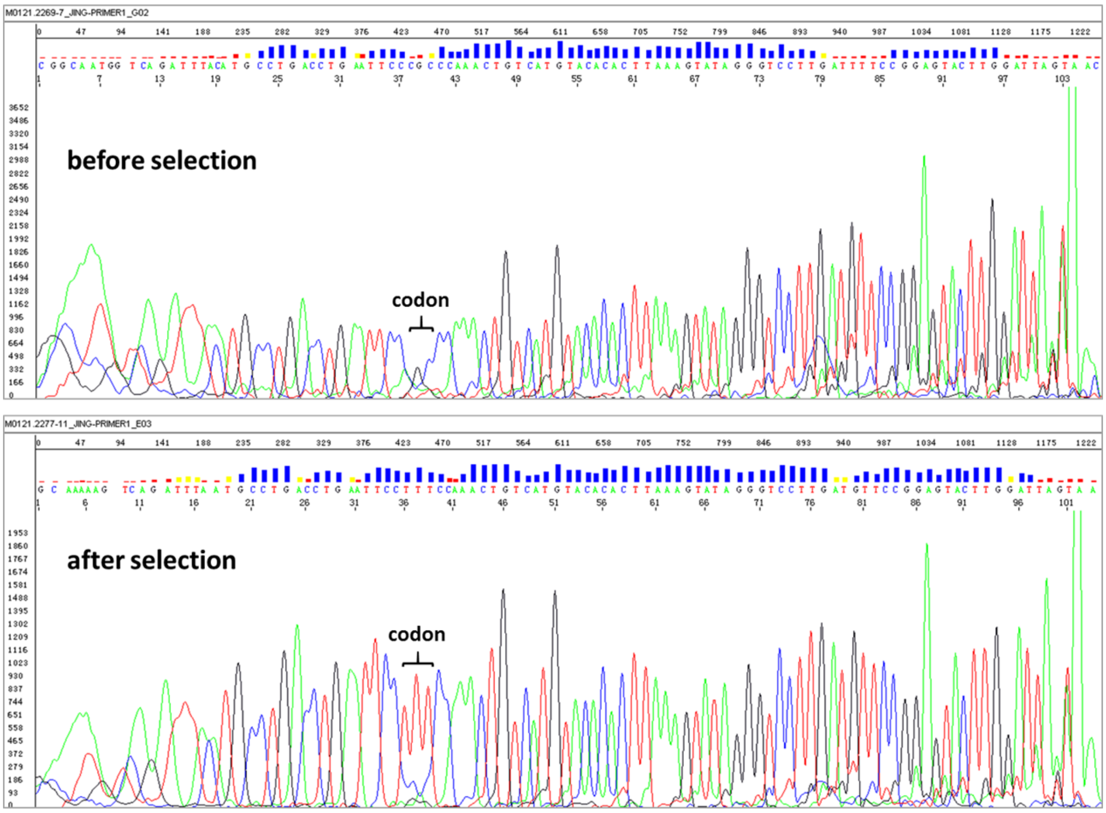

Figure S13: Full images of the sequence result for the equal molar library selection (Figure 6b).

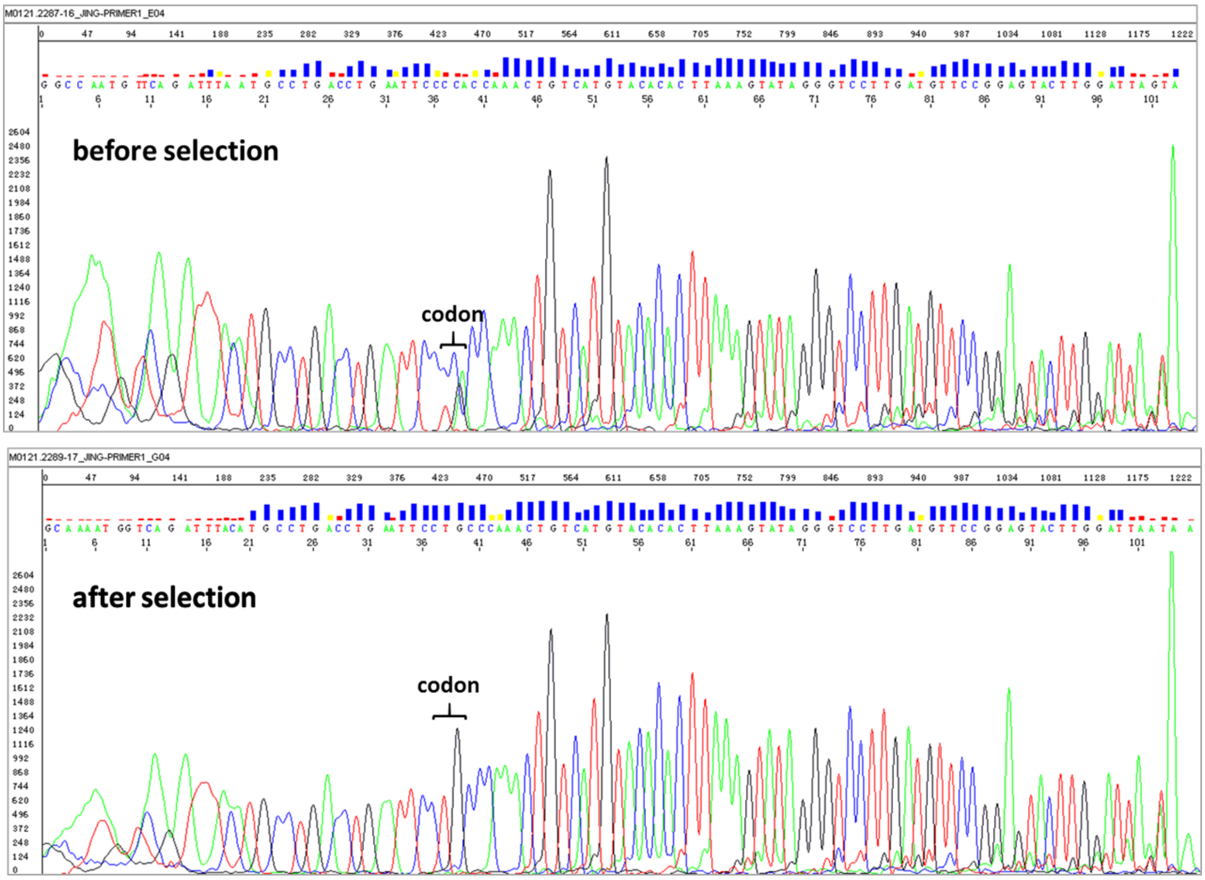

Figure S14: Full images of the sequence result for the 1:100 library selection (Figure 6c).
